# LncRNA OIP5-AS1 upregulates snail expression by sponging miR-34a to promote ovarian carcinoma cell invasion and migration

**DOI:** 10.1186/s40659-020-00315-1

**Published:** 2020-10-22

**Authors:** Xingzhi Jiang, Zhongxue Ye, Yafen Jiang, Wen Yu, Qian Fang

**Affiliations:** 1 Department of Gynaecology, Hwa Mei Hospital, University of Chinese Academy of Sciences, No. 41 Northeast Street, Haishu District, Ningbo, 315000 Zhejiang People’s Republic of China; 2 Ningbo Institute of Life and Health Industry, University of Chinese Academy of Sciences, Ningbo, 315000 Zhejiang People’s Republic of China

**Keywords:** OIP5-AS1, OC, miR-34a, Snail

## Abstract

**Background:**

Although OIP5-AS1 has been characterized as an oncogenic lncRNA in many types of cancer, its role and underlying mechanism in ovarian carcinoma (OC) remains unknown. This study aimed to investigate the role of OIP5-AS1 in OC.

**Methods:**

OC tissues and non-tumor tissues (ovary tissues within 3 cm around tumors) were collected from 58 OC patients (age range 36 to 67 years old, mean age 51.4 ± 5.9 years old). The expression of OIP5-AS1 and snail in paired tissues were determined by RT-qPCR. The interaction between OIP5-AS1 and miR-34a was predicted by IntaRNA2.0 and confirmed by dual luciferase reporter assay. The effects of overexpression of OIP5-AS1 and miR-34a on the expression of snail were analyzed by RT-qPCR and Western blotting. Cell invasion and migration were analyzed by Transwell assay.

**Results:**

We observed that the expression of OIP5-AS1 and snail was upregulated and positively correlated with each other in OC. RNA–RNA interaction analysis showed that OIP5-AS1 might sponge miR-34a. In OC cells, overexpression of OIP5-AS1 resulted in the upregulated expression of snail, while overexpression of miR-34a downregulated the expression of snail. In addition, overexpression of miR-34a reduced the effects of overexpression of OIP5-AS1 on the expression of snail. In cell invasion and migration assay, overexpression of OIP5-AS1 and snail resulted in increased OC cell invasion and migration, while overexpression of miR-34a decreased OC cell invasion and migration. Moreover, overexpression of miR-34a attenuated the effects of OIP5-AS1 overexpression on OC cell invasion and migration.

**Conclusions:**

Therefore, OIP5-AS1 may upregulate snail expression in OC by sponging miR-34a to promote OC cell invasion and migration.

## Background

Ovarian carcinoma (OC) is one of the most common types of malignancy in females for years [1]. In 2018, OC affected more than 22,000 new cases and caused more than 14,000 deaths in the United States [2]. OC accounts for only 2.5% of all cancer cases in women. However, more than 5% of cancer-related deaths are caused by OC, which is mainly due to its low invasiveness and early diagnosis rate [3]. Survival of OC patients is significantly impacted by clinical stages. It has been estimated that more than 93% of OC patients at local stage can live longer than 5 years after initial diagnosis [4]. Therefore, it’s of great clinical values to develop novel early diagnostic markers for OC [5]. However, the pathogenesis of OC is still unclear, which limits the development of diagnostic and therapeutic methods [6].

Snail (zinc finger protein SNAIL) is a zinc finger transcriptional repressor that participates in human cancers through the regulation of epithelial-mesenchymal transition (EMT) [7–9]. In effect, tumor suppressive miRNAs, such as miR-22, miR-137 and miR-34a, can directly target snail to inhibit the metastasis of tumors [10, 11]. It has been well established that miRNAs can interact with other non-coding RNAs, such as long (> 200 nt) non-coding RNAs (lncRNAs) to participate in cancer progression [12]. MiRNAs may serve as the upstream regulators of lncRNAs, or lncRNAs may sponge miRNAs to suppress their functions [12]. As a long noncoding RNA, OPA-interacting protein 5 antisense transcript 1 (OIP5-AS1) transcribed in the opposite direction to OIP5 which located on human chromosome 15q15.1 with 9 exon count [13]. OIP5-AS1 has been characterized as an oncogenic lncRNA in many types of cancer [14, 15]. Our preliminary bioinformatics analysis showed that miR-34a could form multiple base pairs with OIP5-AS1. This study aimed to investigate the role and underlying mechanism of OIP5-AS1 in OC and found that OIP5-AS1 might upregulate snail expression in OC by sponging miR-34a to promote OC cell invasion and migration.

## Methods

### OC patients

OC (primary tumor) tissues and non-tumor tissues (ovary tissues within 3 cm around tumors) were collected from 58 OC patients (serous types, age range 36 to 67 years old, mean age 51.4 ± 5.9 years old). All tissue samples were confirmed by histopathological exam. All these patients were selected from the OC patients admitted to Hwa Mei Hospital, University of Chinese Academy of Sciences from June 2016 to March 2019. Inclusion criteria were: (1) complete medical records; (2) newly diagnosed OC cases; (3) therapies were not initiated before admission. Exclusion criteria were: (1) recurrent OC; (2) transferred from other hospitals; (3) history of other malignancies; (4) other clinical disorders were diagnosed. All the 58 OC patients understood the experimental designed, and they all signed the informed consent. According to the AJCC criteria, there were 9, 11, 17 and 21 cases at clinical stage I–IV, respectively. This study passed the review board of the Ethics Committee of Hwa Mei Hospital, University of Chinese Academy of Sciences. And all experiments were conducted in accordance with the Declaration of Helsinki.

### OC cells and transfections

Human OC cell line UWB1.289 (ATCC, USA) and A2780 (ATCC, USA) were used. Cells were cultivated in the mixture of 48.5% MEGM, 48.5% RPMI-1640 medium and 3% FBS. Cell culture conditions were 37 °C and 5% CO_2_. To perform overexpression experiment, pcDNA3 vectors expressing OIP5-AS1 and snail were constructed by GenePharma (Shanghai, China). Negative control miRNA and miR-34a mimic were synthesized by RIBOBIO (Guangzhou, China). Lipofectamine 2000 (GenePharma) and 10 nM vector or 40 nM miRNA were transfected into 10^6^ cells. Negative control (empty vector or negative control miRNA transfection) and the Control (un-transfected cells) were included. Cells were harvested at 24 h post-transfection for the following experiments.

### RNA–miRNA interaction prediction

OIP5-AS1 sequence (long sequence) and miR-34a sequence (short sequence) were inputted into IntaRNA 2.0 (http://rna.informatik.uni-freiburg.de/IntaRNA/Input.jsp) to predict the interaction between OIP5-AS1 and miR-34a. Snail and miR-34a sequence (short sequence) were inputted into RNA22Sites (https://cm.jefferson.edu/rna22/Interactive/RNA22Controller) to predict the interaction between snail and miR-34a. Default parameters were used.

### Dual luciferase reporter assay

OIP5-AS1 luciferase vector (OIP5-AS1 sequence followed by the sequence of luciferase gene) was constructed with pGL3 vector (Promega Corporation). OIP5-AS1 luciferase vector combined with NC miRNA (NC group) or miR-34a mimic (miR-34a group) was transfected into UWB1.289 cells using lipofectamine 2000. Luciferase activity was measured 48 h later.

### RNA extractions

Non-tumor and OC tissues weighted from 0.018 to 0.023 g. Tissue specimens (0.15 g) from each tissue were ground in liquid nitrogen. UWB1.289 and A2780 cells were harvested and counted. The ground tissues and 10^6^ cells were mixed with 1 ml Trizol (Invitrogen, USA) to extract total RNAs. To harvest miRNAs, 85% ethanol was used to precipitate and wash RNA samples.

### RT-qPCR

DNase I was used to digest all RNA samples at 37 °C for 90 min to remove genomic DNAs. To measure the expression levels of OIP5-AS1 and snail, QuantiTect Reverse Transcription Kit (QIAGEN) and SYBR Green Master Mix (Bio-Rad) were used to perform reverse transcriptions (RTs) and prepare qPCR mixtures, respectively. GAPDH was used as the endogenous control. To detect the expression of miR-34a, All-in-One™ miRNA qRT-PCR Detection Kit (Genecopoeia, Guangzhou, China) was used to perform all RTs and qPCR reactions with U6 as endogenous control. Three replicate reactions were included in each experiment. Primer sequences were: OIP5-AS1-F: 5′-TGCGAAGATGGCGGAGTAAG-3′,

OIP5-AS1-R: 5′-TAGTTCCTCTCCTCTGGCCG-3′;

Snail-F: 5′-CTGGCAGCCATCCCACCTCC-3′;

Snail-R: 5′-ACAGGGAGGTCAGCTCTGCCA-3′;

GAPDH-F: 5′-CTCAGACACCATGGGGAAGGTGA-3′;

GAPDH-R: 5′-ATGATCTTGAGGCTGTTGTCATA-3′.

MiR-34a was 5′-TGGCAGTGTCTTAGCTGGT-3′. U6 forward primer and universal reverse primer were from the kit. PCR reactions conditions were: 95 °C for 1 min, followed by 40 cycles of 95 °C for 10 s and 60 °C for 56 s. All Ct values were normalized using the 2^−ΔΔCT^ method. It was worth noting that β-actin was also used as an internal control, and similar results were obtained.

### Western blotting

UWB1.289 and A2780 cells were counted, and 10^6^ cells were mixed with 1 ml RIPA (GenePharma) to extract total proteins. Proteins were boiled in water for 5 min, and electrophoresis was performed using 10% SDS-PAGE gel. PVDF membranes were used in gel transfer, and PBS containing 5% non-fat milk was used to block at room temperature for 2 h. GAPDH (1:900, ab37168, Abcam) and snail (1:900, ab53519, Abcam) primary antibodies were used in the first blot, which was performed at 4 °C overnight. HRP goat anti-rabbit (IgG) (1:1000; ab6721; Abcam) secondary antibody was used in the second blot, which was performed at room temperature for 2 h. RapidStep™ ECL detection reagent (EMD Millipore) was dropped onto the membranes to develop signals. Gray values were analyzed using Image J v1.46 software.

### Cell invasion and migration assays

UWB1.289 and A2780 cells were harvested respectively, and 3 × 10^4^ cells were mixed with 1 ml aforementioned cell culture medium (non-serum) to prepare single cell suspensions. Transwell chambers (Corning^®^ Transwell^®^, Sigma-Aldrich) were used. 0.1 ml cell suspension was added into the upper Transwell chamber, and the lower chamber was filled with aforementioned cell culture medium containing 20% FBS to induce cell migration and invasion. Matrigel (Millipore) was used to coat the upper chamber membranes at 37 °C for 6 h before invasion assay. Cells were cultivated at 37 °C for 12 h. After staining using 1% crystal violet (Sigma-Aldrich), cells were counted under a light microscope.

### Data analysis

All experiments included 3 biological replicates, and data were expressed as the mean values. Paired t-test and ANOVA (one-way) combined with Tukey test were used to explore differences between two tissue types (OC and non-tumor) and among multiple cell groups, respectively. Correlations were analyzed using linear regression. Patients were divided into high and low expression levels of OIP5-AS1 or snail groups (n = 29). The correlations between the expression levels of OIP5-AS1 or snail in OC tissues and patients’ clinical data were analyzed by Chi-squared test. *p* < 0.05 was considered as statistically significant.

## Results

### The expression of OIP5-AS1 and snail were upregulated in OC

**Fig. 1:**
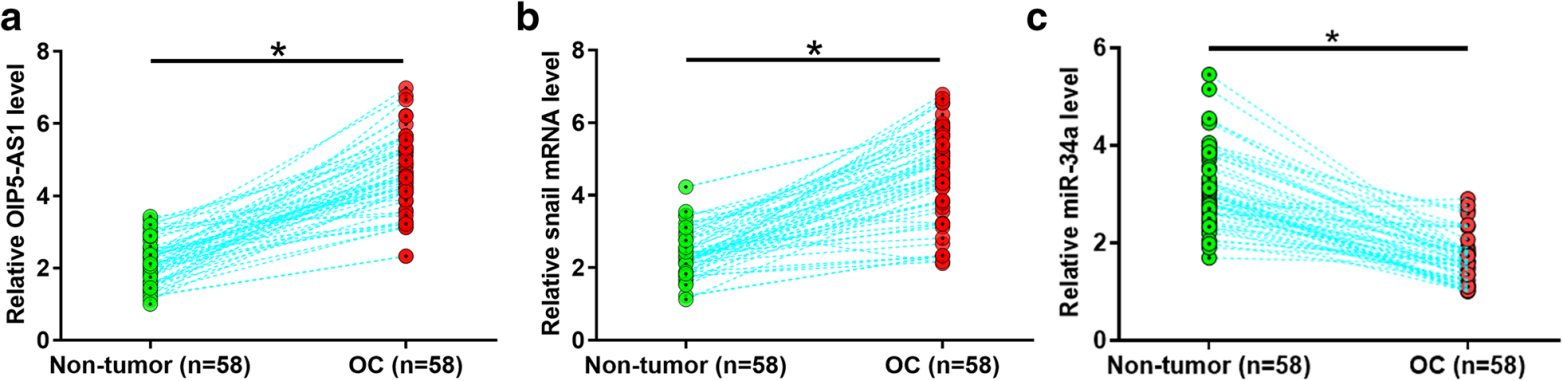
The expression of OIP5-AS1 and snail were upregulated in OC. Expression levels of OIP5-AS1 (**a**) and snail (**b**) in non-tumor and OC tissues were measured by qRT-PCR. Expression data between two tissue types were compared by paired t-test. QRT-PCR was performed 3 times and data were expressed as mean values, *p < 0.05

To investigate the expression of OIP5-AS1 and snail in OC tissue, RT-qPCR was used. It was observed that the expression of OIP5-AS1 (Fig. 1a) and snail (Fig. 1b) were significantly increased in OC tissues, while miR-34a (Fig. 1c) was decreased compared with non-tumor tissues (*p* < 0.05). Chi-squared test showed that the expression levels of OIP5-AS1 were significantly correlated with cancer grades but not cancer stages, age and BMI (Table 1). The expression levels of snail were significantly correlated with cancer grade and stage but not age and BMI (Table 2).

**Table 1:** Correlation between levels of OIP5-AS1 expression and patients clinical factors

Items	Groups	Cases	High-expression	Low-expression	χ^2^	p value
Age	> 50 (years)	28	12	16	1.10	2.29
	<= 50 (years)	30	17	13		
Stages	I	9	2	7	5.00	0.14
	II	11	4	7		
	III	17	11	6		
	IV	21	12	9		
Grade	Low	32	10	22	10.04	0.02
	High	26	19	7		
BMI	> 24	23	11	12	0.07	0.79
	<= 24	35	18	17

**Table 2:** Correlation between levels of snail mRNA expression and patients clinical factors

Items	Groups	Cases	High-expression	Low-expression	χ^2^	p value
Age	>50 (years)	28	11	17	2.49	0.11
	<= 50 (years)	30	18	12		
Stages	I	9	3	6	8.32	0.04
	II	11	2	9		
	III	17	10	7		
	IV	21	14	7		
Grade	Low	32	8	24	17.85	0.01
	High	26	21	5		
BMI	> 24	23	10	13	0.65	0.42
	<= 24	35	19	16		

### The expression of OIP5-AS1 and snail were positively correlated in OC

**Fig. 2:**
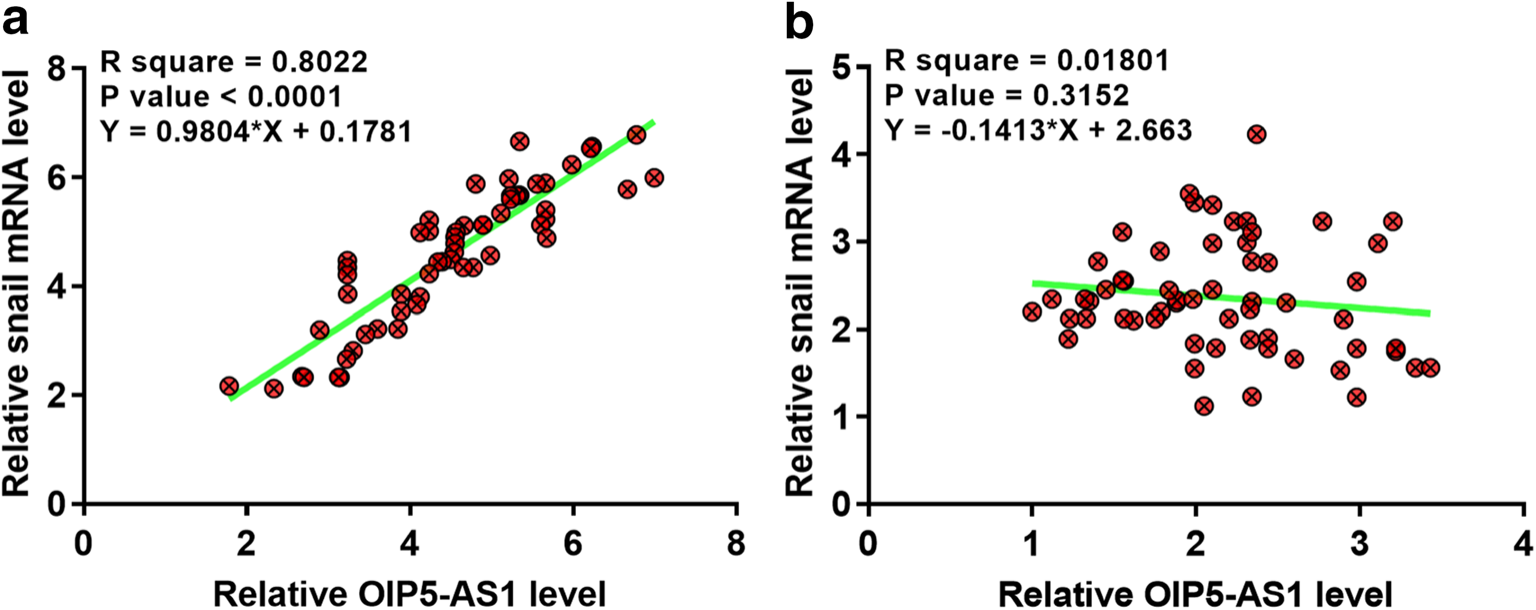
The expression of OIP5-AS1 and snail were positively correlated in OC. Correlation between OIP5-AS1 (**a**) and snail (**b**) was analyzed by linear regression

Correlation between OIP5-AS1 and snail was analyzed by performing linear regression. In OC tissues, the expression of OIP5-AS1 and snail was significantly and positively correlated (Fig. 2a). However, no close correlation between the expression of OIP5-AS1 and snail was observed across non-tumor tissues (Fig. 2b).

### OIP5-AS1 might sponge miR-34a to upregulate snail

**Fig. 3:**
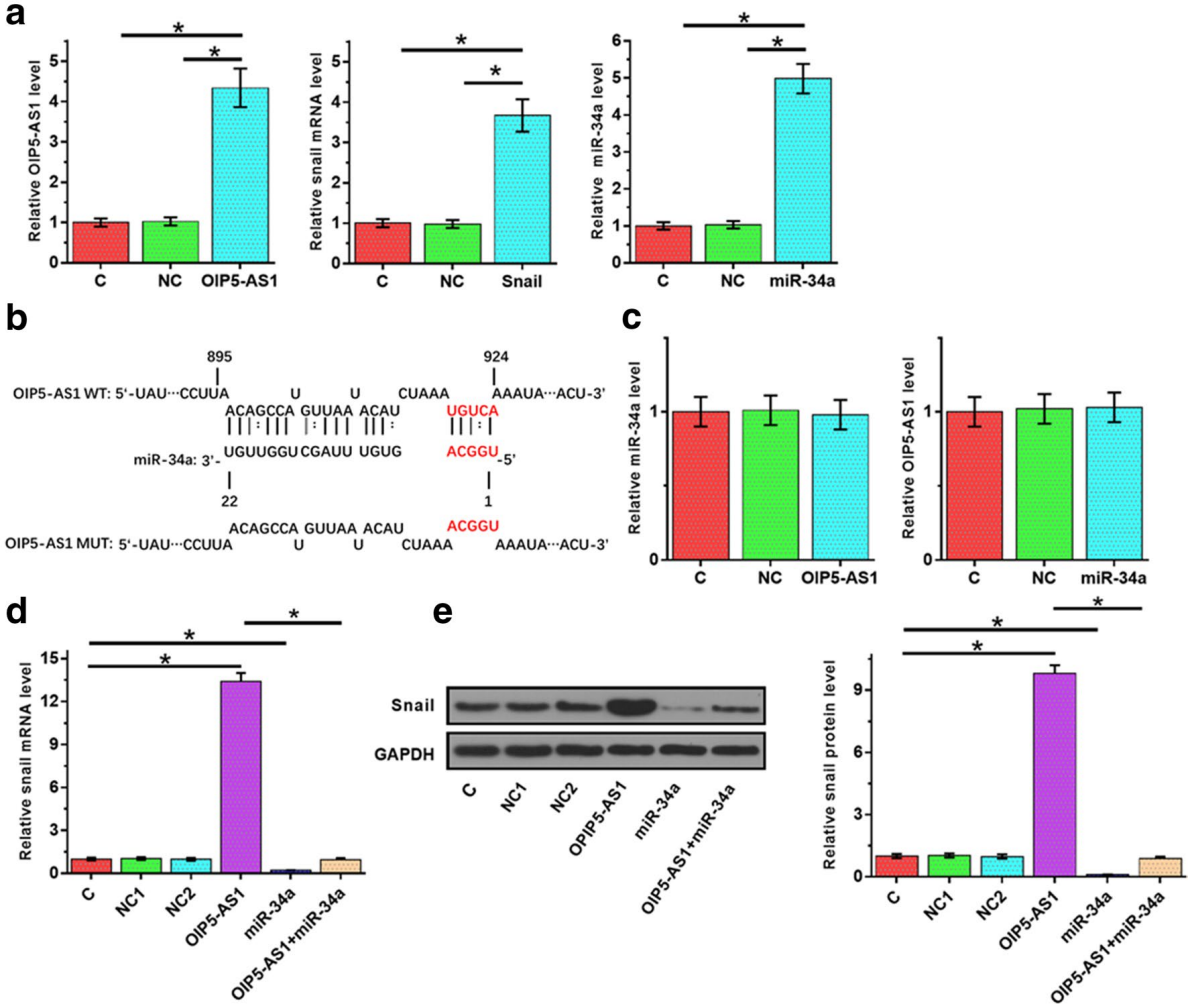
OIP5-AS1 might sponge miR-34a to upregulate snail. To investigate the interaction between OIP5-AS1, miR-34a and snail, OIP5-AS1 expression vector, miR-34a mimic and snail expression vector were transfected into UWB1.289 cells. At 24 h post-transfection, the expression levels of OIP5-AS1, miR-34a and snail were measured by qRT-PCR (**a**). The interaction between miR-34a and OIP5-AS1 was predicted by IntaRNA (**b**). The interaction between OIP5-AS1 and miR-34a was analyzed by qRT-PCR (**c**). The effects of OIP5-AS1and miR-34a on the expression of snail were analyzed by qRT-PCR (**d**) and western blotting (**e**), respectively. Western blotting and qRT-PCR were repeated 3 times, and the mean values were expressed. NC1, empty pcDNA3 transfection; NC2, negative control miRNA transfection; *p < 0.05

To investigate the interaction among OIP5-AS1, miR-34a and snail, OIP5-AS1 and snail expression vectors as well as miR-34a mimic were transfected into UWB1.289 cells. At 24 h post-transfections, the expression of OIP5-AS1 and snail was significantly increased (Fig. 3a, *p* < 0.05). RNA–miRNA interaction analysis showed that miR-34a might bind with OIP5-AS1 (Fig. 3b), and miR-34a could directly target snail (Additional file 1: Figure S2). Dual luciferase activity assay was performed to further confirm the interaction between OIP5-AS1 and miR-34a. Compared with cells transfected with OIP5-AS1 and NC miRNA (NC group), cells transfected with OIP5-AS1 and miR-34a (miR-34a group) showed significantly lower luciferase activity, which confirmed the interaction between OIP5-AS1 and miR-34a (Additional file 2: Figure S1). However, overexpression of OIP5-AS1 and miR-34a did not affect the expression of each other (Fig. 3c). Overexpression of OIP5-AS1 upregulated the expression of snail, while overexpression of miR-34a downregulated the expression of snail. In addition, overexpression of miR-34a reduced the effects of overexpression of OIP5-AS1 on both mRNA (Fig. 3d, *p *< 0.05) and protein level (Fig. 3e, *p *< 0.05). We speculated that overexpression of OIP5-AS1 upregulated expression of snail by sponging miR-34a. Meanwhile, we found the same results at A2780 cell (Additional file 3: Figure S3).

### OIP5-AS1 promoted UWB1.289 and A2780 cell invasion and migration

**Fig. 4:**
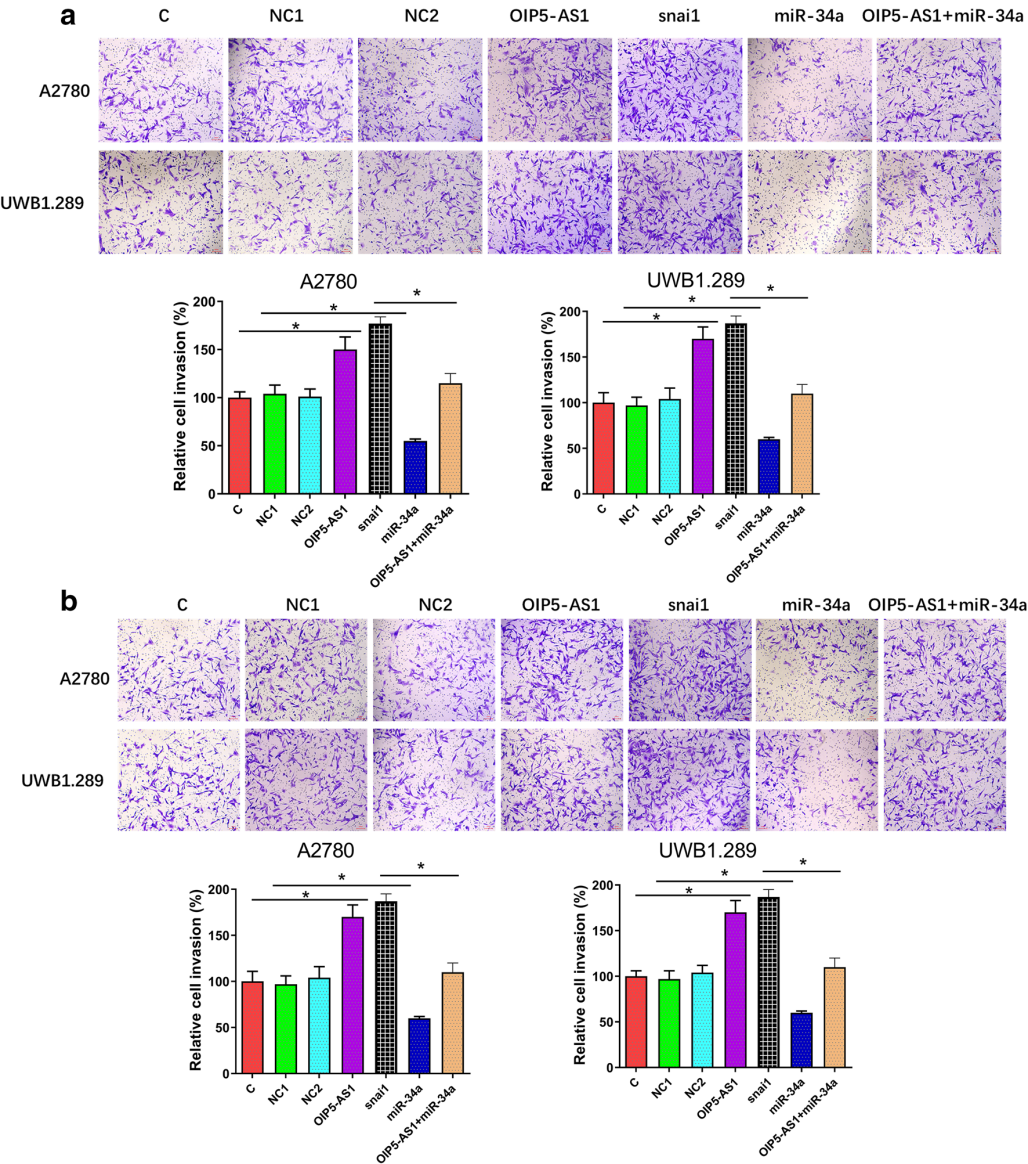
OIP5-AS1 promoted UWB1.289 and A2780 cell invasion and migration. Cell invasion (**a**) and migration (**b**) data were analyzed by performing ANOVA (one-way) and Tukey-test. Transwell invasion and migration assays were repeated 3 times, and mean values were expressed (*p < 0.05)

Cell invasion and migration data were analyzed by performing ANOVA (one-way) and Tukey test. Compared with C and NC groups, overexpression of OIP5-AS1 and snail resulted in increased cell invasion and migration, while overexpression of miR-34a decreased the rates of cell invasion (Fig. 4a) and migration (Fig. 4b). Moreover, overexpression of miR-34a attenuated the effects of overexpression of OIP5-AS1 (*p *< 0.05).

## Discussion

LncRNAs are abnormally expressed in a variety of human malignant tumors, and these abnormally expressed LncRNAs can be used as biomarkers for early clinical diagnosis. Recently, it has found that OIP5-AS1, as a novel lncRNA, is abnormally expressed in multiple tumors and can be used as a clinical biomarker for liver hepatocellular carcinoma and adenocarcinoma [16, 17]. However, its expression pattern and biological role in OC have not been well studied. In this study, we investigated the role of OIP5-AS1 in OC, and found that OIP5-AS1 was upregulated in OC. OIP5-AS1 may sponge miR-34a, and then upregulate the expression of its downstream target snail, thereby promoting invasion and migration of OC cells.

OIP5-AS1 has been characterized as an oncogenic lncRNA in many types of cancer [16–18]. In lung cancer, OIP5-AS1 targets miR-448 and Bcl-2 to regulate the proliferation and apoptosis of cancer cells [14]. OIP5-AS1 was reported to participate in the regulation of radio-resistance of colorectal cancer cells by interacting with miR-369-3p and DYRK1A [15]. In liver cancer, OIP5-AS1 promotes the proliferation, EMT progress and metastasis by downregulating ZEB1 and upregulating miR-186a-5p [19]. To our best knowledge, the role of OIP5-AS1 in OC has not been well studied. This study is the first to report the upregulation of OIP5-AS1 in OC, and overexpression of OIP5-AS1 was found to increase the invasion and migration of OC cells. Our study demonstrated the oncogenic role of OIP5-AS1 in OC.

MiR-34a is a mature tumor suppressor miRNA, which has been confirmed in a variety of cancers, such as hepatocellular carcinoma and colorectal cancer [20, 21]. Moreover, miR-34a is reported to be frequently downregulated in OC, and the overexpression of miR-34a can inhibit cancer progression by suppressing the expression of many oncogenic genes, such as snail [11, 22]. In this study, we also observed that the expression of miR-34a was downregulated in OC, while invasion and migration of OC cells were inhibited after miR-34a overexpression. In addition, overexpression of miR-34a resulted in the downregulation of snail expression. Therefore, our data confirmed that miR-34a could target snail to inhibit the invasion and migration of OC cells.

It is known that miR-34a targets snail to reduce the expression of snail at both mRNA and protein levels [11]. In this study, we found that miR-34a could directly interact with OIP5-AS1, but overexpression of miR-34a and OIP5-AS1 did not affect the expression of each other. Therefore, OIP5-AS1 may not be a target of miR-34a. Besides, being the target of miRNAs, lncRNAs may also sponge miRNAs to suppress their roles [23]. In this study, overexpression of OIP5-AS1 was found to reduce the inhibitory effects of miR-34a on the snail expression and OC cell invasion and migration. Therefore, we speculated that OIP5-AS1 might sponge miR-34a to upregulate oncogenic snail expression, thereby promoting OC cell invasion and migration. However, studies are needed to further test this possibility.

## Conclusion

In conclusion, OIP5-AS1 is overexpressed in OC and may sponge miR-34a to upregulate snail expression, there by promoting OC cell invasion and migration.

## Supplementary information

**Additional file 1: Figure S2.** The interaction between miR-34a and snail was predicted by IntaRNA.

**Additional file 2: Figure S1.** Dual luciferase activity assay was performed by using lipofectamine 2000 to transfect OIP5-AS1 luciferase vector combined with NC miRNA (NC group) or miR-34a mimic (miR-34a group) into UWB1.289 cells. Luciferase activity was measured 48 h later. This experiment was repeated 3 times and mean values were expressed (*p < 0.05).

**Additional file 3: Figure S3.** To investigate the interaction between OIP5-AS1, miR-34a and snail, OIP5-AS1 expression vector, miR-34a mimic and snail expression vector were transfected into A2780 cells. The effects of OIP5-AS1and miR-34a on the expression of snail were analyzed by qPCR (A) and western blot (B), respectively. Western blot and qPCR were repeated 3 times and the mean values were expressed. NC1, empty pcDNA3 transfection; NC2, negative control miRNA transfection; *p < 0.05.

## Data Availability

The datasets generated and/or analyzed during the current study are not publicly available due research design, but are available from the corresponding author on reasonable request.

## Abbreviations

OC: Ovarian carcinoma
EMT: Epithelial-mesenchymal transition
lncRNA: Long non-coding RNA
AJCC: American Joint Committee on Cancer
