# Supplementary figures and images for: LncRNA OIP5-AS1 upregulates snail expression by sponging miR-34a to promote ovarian carcinoma cell invasion and migration
Source: Biol Res. 2020 Oct 22;53:49. doi: 10.1186/s40659-020-00315-1 (PMC7579860; doi:10.1186/s40659-020-00315-1)

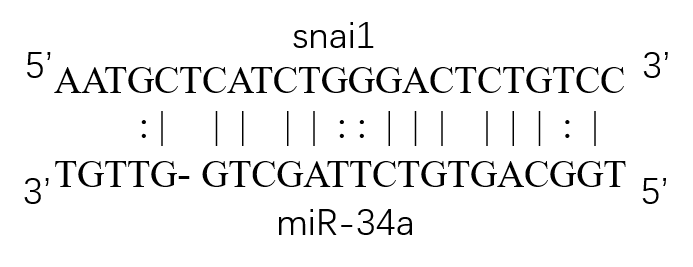

Supplement: Supplementary file 1 — Additional file 1: Figure S2. The interaction between miR-34a and snail was predicted by IntaRNA. [file 40659_2020_315_MOESM1_ESM.tif]

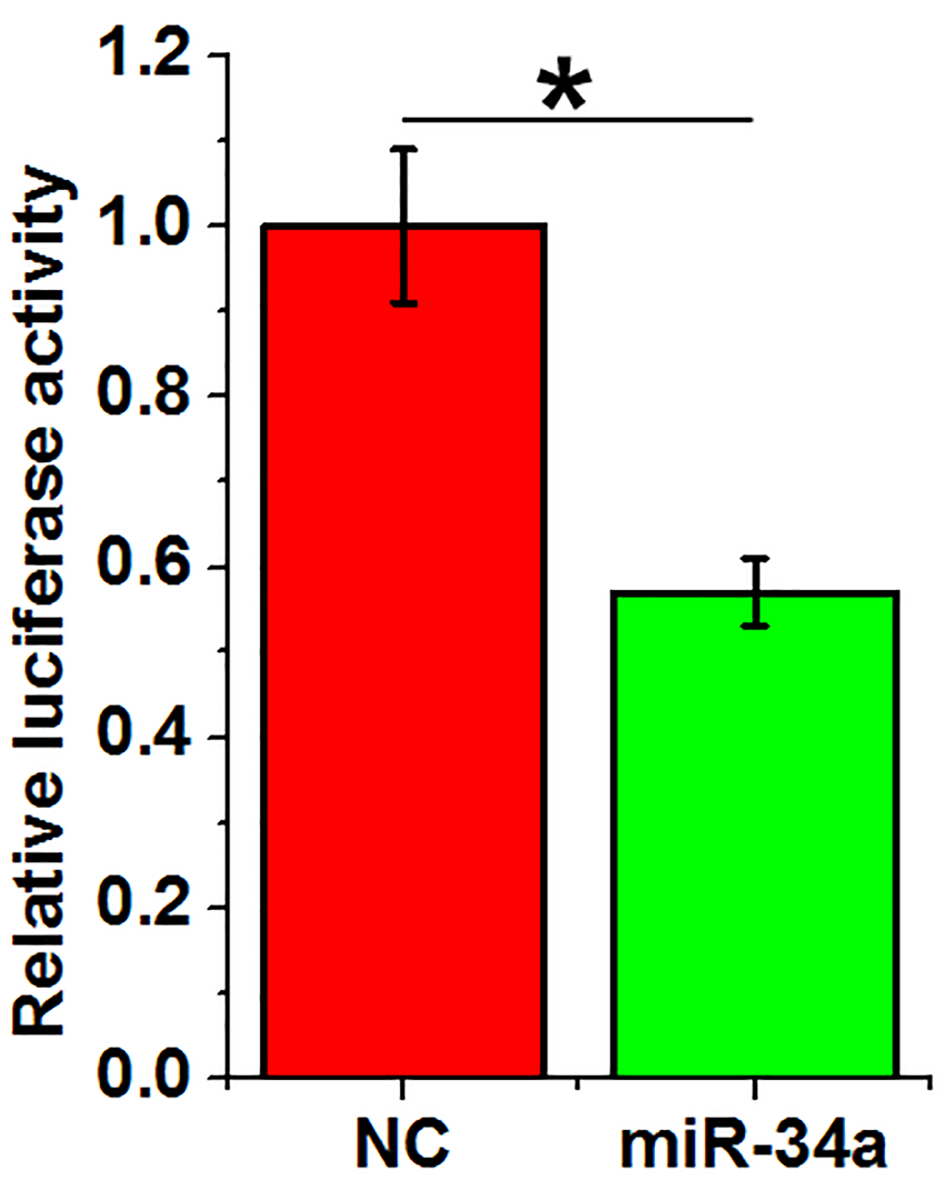

Supplement: Supplementary file 2 — Additional file 2: Figure S1. Dual luciferase activity assay was performed by using lipofectamine 2000 to transfect OIP5-AS1 luciferase vector combined with NC miRNA (NC group) or miR-34a mimic (miR-34a group) into UWB1.289 cells. Luciferase activity was measured 48 h later. This experiment was repeated 3 times and mean values were expressed (*p < 0.05). [file 40659_2020_315_MOESM2_ESM.tif]

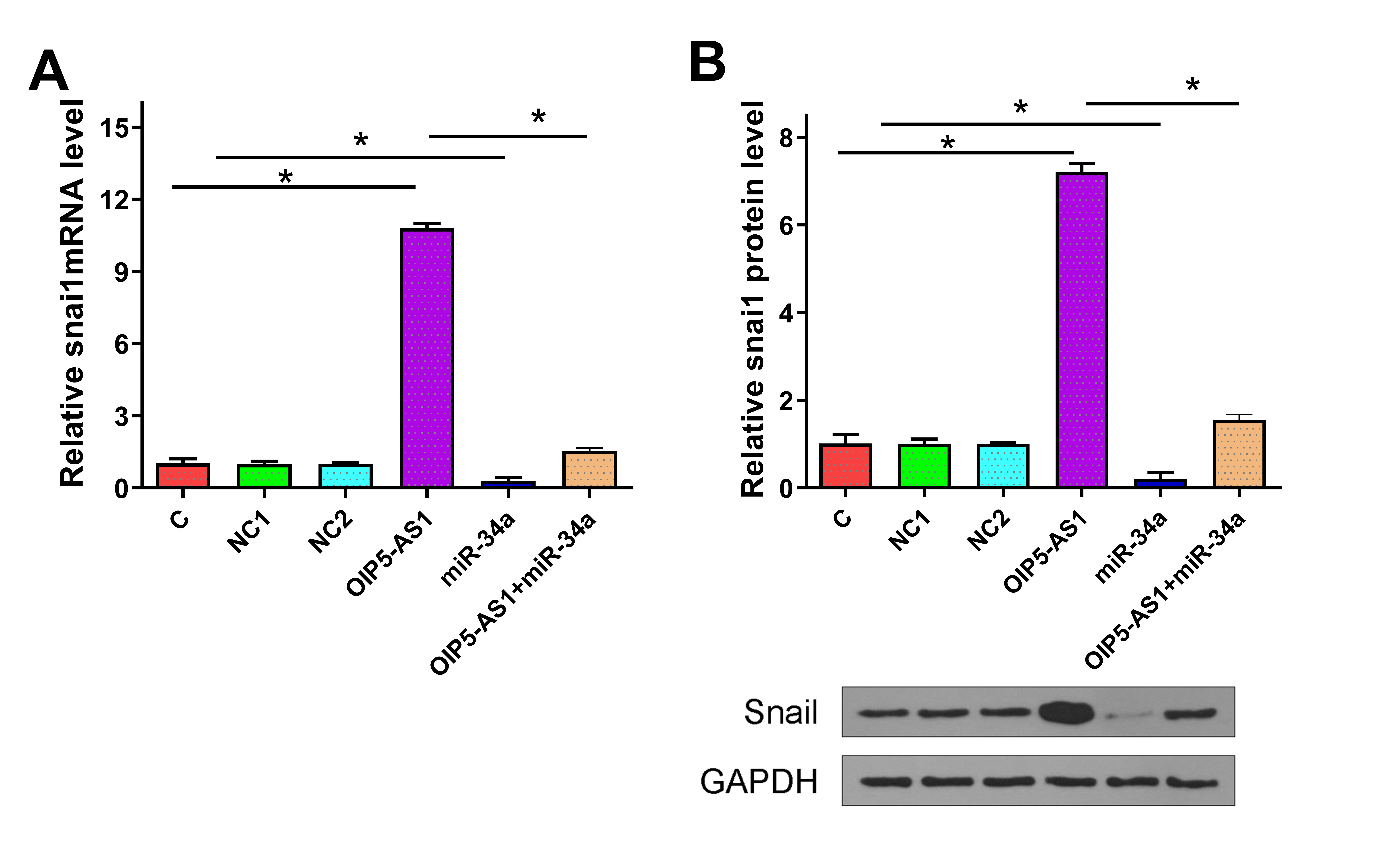

Supplement: Supplementary file 3 — Additional file 3: Figure S3. To investigate the interaction between OIP5-AS1, miR-34a and snail, OIP5-AS1 expression vector, miR-34a mimic and snail expression vector were transfected into A2780 cells. The effects of OIP5-AS1and miR-34a on the expression of snail were analyzed by qPCR (A) and western blot (B), respectively. Western blot and qPCR were repeated 3 times and the mean values were expressed. NC1, empty pcDNA3 transfection; NC2, negative control miRNA transfection; *p < 0.05. [file 40659_2020_315_MOESM3_ESM.tif]
